# Single-Cell Analysis of Thymocyte Differentiation: Identification of Transcription Factor Interactions and a Major Stochastic Component in αβ-Lineage Commitment

**DOI:** 10.1371/journal.pone.0073098

**Published:** 2013-10-01

**Authors:** Amine Boudil, Lamia Skhiri, Serge Candéias, Valérie Pasqualetto, Agnès Legrand, Marie Bedora-Faure, Laetitia Gautreau-Rolland, Benedita Rocha, Sophie Ezine

**Affiliations:** 1 Institut National de la Santé et de la Recherche Médicale, Unité 1020, and Université Paris Descartes, Unité Mixte de Recherche, Paris, France; 2 Institut de Recherches en Technologies et Sciences pour le Vivant, Laboratoire de Chimie et Biologie des Métaux, UMR 549 Centre national de la recherche scientifique, Université Joseph Fourier, Grenoble, France; Department of Immunology, China

## Abstract

T cell commitment and αβ/γδ lineage specification in the thymus involves interactions between many different genes. Characterization of these interactions thus requires a multiparameter analysis of individual thymocytes. We developed two efficient single-cell methods: (i) the quantitative evaluation of the co-expression levels of nine different genes, with a plating efficiency of 99–100% and a detection limit of 2 mRNA molecules/cell; and (ii) single-cell differentiation cultures, in the presence of OP9 cells transfected with the thymus Notch1 ligand DeltaL4. We show that during T cell commitment, Gata3 has a fundamental, dose-dependent role in maintaining Notch1 expression, with thymocytes becoming T-cell-committed when they co-express Notch1, Gata3 and Bc11b. Of the transcription factor expression patterns studied here, only that of Bcl11b was suggestive of a role in Pu1 down-regulation. Individual thymocytes became αβ/γδ lineage-committed at very different stages (from the TN2a stage onwards). However, 20% of TN3 cells are not αβ/γδ-lineage committed and TN4 cells comprise two main subpopulations with different degrees of maturity. The existence of a correlation between differentiation potential and expression of the pre-TCR showed that 83% of αβ-committed cells do not express the pre-TCR and revealed a major stochastic component in αβ-lineage specification.

## Introduction

In the thymus, T lymphocytes develop from precursor cells that do not express CD4, CD8 or CD3. These triple-negative (TN) cells undergo several successive differentiation stages. The early thymus progenitors (ETPs) are CD44^+^c-Kit^+^IL-7R^−^CD25^−^ and are still able to generate myeloid cells, natural killer (NK) cells and rare B cells. These precursors upregulate c-Kit, IL-7R and CD25 and generate the TN2a population. The latter cells have lost B cell potential and, when compared with the ETP population, are poorly capable of generating NK cells (thus indicating significant T cell commitment). However, full T cell commitment is only achieved when TN2a thymocytes downregulate the expression of c-Kit and IL-7R to become TN2b cells. The TN2b populations then lose CD44 expression to yield TN3 thymocytes – the most abundant TN population. It is believed that the majority of TCR-λ and TCR-β complete rearrangements occur during this differentiation phase. Successful rearrangements enable TN3a thymocytes to pass the pre-TCR/γδ check point and become TN3b thymocytes. This selection step induces a major proliferative burst and the upregulation of CD27, which reportedly discriminates between selected and non-selected cells. The TN3b thymocytes further progress to the TN4 stage (where expression of CD25 is lost) and eventually co-express CD4 and CD8αβ heterodimers to become double-positive (DP) thymocytes. It is known that all TCR-αβ^+^ CD4^+^ or CD8αβ^+^ thymocytes pass through an intermediate DP differentiation stage. In contrast, although the majority of γδ lineage cells do not transit through a DP differentiation phase, they reportedly emerge at various differentiation stages (from TN3 through to DP thymocytes).

Although T cell commitment is dependent on the master regulator Notch1, the Gata3 and Bcl11b transcription factors (TFs) must associate to Notch1 to induce this commitment [1]. The lack of either Notch1 or its target gene Gata3 induces a similar, early block in TN1 cell differentiation [2], [3]. Investigations of Bcl11b's role have yielded contradictory results [4], [5], [6], [7]. Early studies of Bcl11b^−/−^ thymocytes reported an increase in TN3 CD44^−^CD25^+^ thymocyte counts, massive apoptosis and elevated TCR-γδ generation [7]. In contrast, both Bcl11b inactivation in the fetal liver and bone marrow (BM) progenitors in culture on OP9DL1 cells induced a TN2a differentiation arrest [4], [5], [6], with Bcl11b-deficient precursors acquiring the self-renewal capacities that are characteristic of stem cells. Other critical TFs downstream of Notch1 signaling (Hes1 and Tcf-1) also control early T-lineage development [8], [9]. However, it is not clear how these TFs interact. Although the expression of non-T master genes also declines, it is not known how this is induced or whether total repression of the non-T master genes is required for T cell commitment.

T-cell-committed precursors eventually become committed to the αβ or γδ lineage. Despite significant research efforts, there is still much debate as to (i) the precise differentiation stages at which commitment occurs and is completed and (ii) the relative roles of a particular combination of master genes (the so-called “stochastic model”) versus TCR-γδ/pre-TCR signaling/signal strength (the so-called “ instructive model”) (reviewed in [10], [11]). In support of the stochastic model, a fraction of TN2 thymocytes thought not to express either signaling complex is already lineage-committed [12] and intrathymically injected fetal TN2 IL-7R^high^ cells generate predominantly TCR-γδ cells [13]. Lastly, only about half of TN2 cells express *Sox13*, which reportedly may be required for γδ lineage commitment [14]. However, the predominant generation of TCR-γδ cells by TN2 IL-7R^high^ fetal thymocytes could be specific for the fetal thymus, which exports TCR-γδ cells (but not TCR-αβ cells) before birth [15]. Given that TCR-γ rearrangements are IL-7-dependent, these TN2 cells could be a minor fetal subset already expressing the TCR-γδ.

The opposing view attributes lineage commitment exclusively to TCR-γδ/pre-TCR-dependent signals and questions the precise differentiation stage at which final commitment occurs. Hence, thymocytes could be “diverted” from their lineage choice by differences in TCR signal strength, with strong TCR-γδ signals inducing a γδ lineage and weaker pre-TCR signals inducing αβ lineage choices [16], [17]. Overall, the lineage choice may be flexible until TN thymocytes differentiate into either DP cells (for the αβ lineage) or CD24^−^ TCR ^+^ CD4^−^CD8^−^ populations (for the γδ lineage) [18]. Lastly, it has been claimed that the presence of the pre-TCR reduces the proportion of TCR-γδ^+^ cells expressing out-of-frame TCRβ rearrangements – indicating that pre-TCR signaling has deviated TN cells towards the TCR-αβ lineage [19].

The debate between stochastic and instructive models has not been resolved, since methodological aspects of the studies used to support each hypothesis have been criticized. The plating efficiency of single cells in OP9DL1 differentiation cultures (used in experiments favoring the stochastic model) was low, since 50% of the TN2 thymocytes and 73% of the TN3 thymocytes did not generate progeny [12]. Plating efficiencies were even lower in experiments supporting instructive models, since 96% of the plated single cells either did not grow in culture or deviated to alternative lineages [18]. These low plating efficiencies cast doubt on whether these differentiation behaviors truly reflect the properties of the cell populations studied. Lastly, the evaluation of the role of pre-TCR signaling in deviating T cells towards the TCR-αβ lineage could be biased, since 60% of the studied TCR-γδ cells (i.e. those lacking detectable TCRB rearrangements or with only mono-allelic TCRB rearrangements) were excluded *a priori* as PCR failures. However, excluded samples were not tested for the presence of the b locus in a germ-line configuration and it has never shown that all TCR-γδ cells have bi-allelic TCRB rearrangements.

Overall, the conflicting results mentioned above highlight several of the limitations of previous experimental approaches. Gene ablation frequently has many effects on cell behavior and so it may be difficult to identify a given gene's precise role in each particular pathway/differentiation step. Since the role of each TF is known to be critically dependent on its concentration, over-expression studies may reveal roles or activate pathways that do not reflect the TF's behavior at physiological concentrations. All such studies focus on the behavior of a single gene and thus are unlikely to reflect the complexity of T cell differentiation, which depends on interactions between many genes in each individual cell. Lastly, so-called “lineage tracers” for the expression of both the δ chain and the pTα have been developed but the results were disappointing. The lineage tracers were expressed well upstream of the expression of the respective genes [20], [21] and thus failed to trace the respective lineages.

These problems could be resolved by combining (i) efficient single-cell methods for the accurate quantification of gene expression and gene association with (ii) single-cell differentiation cultures with a high plating efficiency. Here, we describe our development of this type of method. We created a genetic profiling technique that simultaneously quantifies the absolute number of mRNAs for each of the nine TFs involved in thymocyte differentiation (along with Cd3ε, Rag1 and pTα) in each individual cell. Importantly, all the steps in this method have been validated; the method has a plating efficiency of 99%–100% and can reliably detect as few as 2 mRNA molecules per cell [22]. Furthermore, we combined this method with efficient single-cell differentiation cultures by stimulating individual precursors with the OP9 cell line transfected with the thymus Notch1 ligand DL4 [23]. The use of DL4 (rather than DL1) substantially increased our plating efficiency and enabled us to characterize the kinetics of αβ/γδ lineage specification more accurately. Overall, the combination of these two approaches allowed us to correlate individual thymocyte differentiation potential with its co-expression of several genes. Our results reveal how different TFs associate during T cell commitment and show that 83% of αβ-lineage-committed thymocytes do not express the pre-TCR.

## Results

### Cell populations and single-cell genetic profiling

To study T cell commitment and αβ/γδ lineage specification, lineage-negative (CD8^−^CD3ε^−^B220^−^Mac1^−^CD19^−^CD11c^−^NK1.1^−^CD11b^−^DX5^−^) TN thymocyte progenitors were subdivided according to their c-Kit/CD44/CD25/CD27 profile into subpopulations with differing differentiation potentials: ETPs, TN2a and b, TN3a and b and TN4 (**Fig.** **S1**). Individual cells from each subset were sorted in order to determine (i) the proportion of cells expressing each gene of interest (i.e. the “expression frequency”), (ii) the number of mRNA copies per cell for each expressed gene (i.e. the expression/transcription level) and (iii) the gene combinations expressed by each individual cell (i.e. gene co-expression).

**Figure 1:**
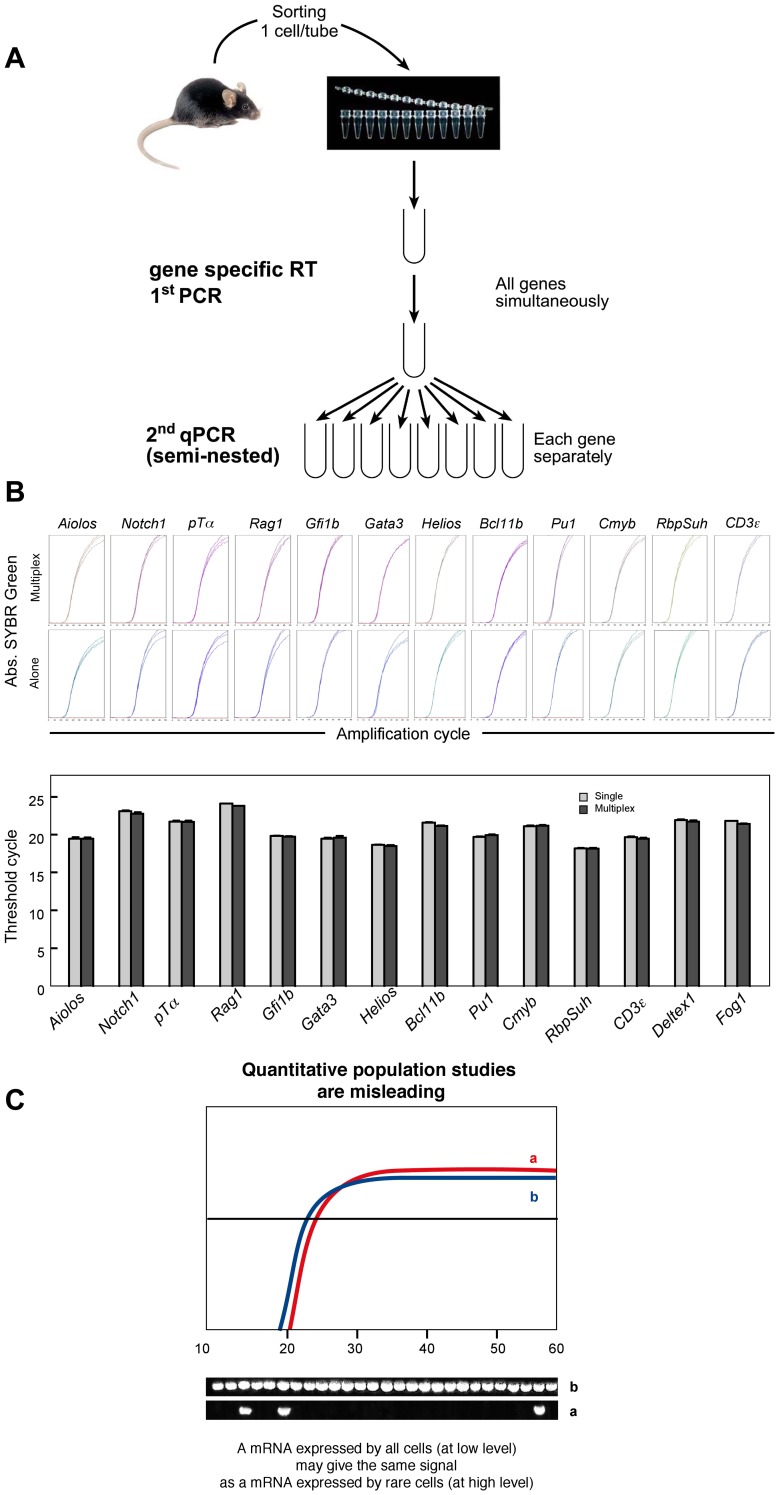
Single-cell quantitative gene expression profiling. (**A**) Overall strategy. (**B**) Validation of the primer pairs used to quantify gene expression. Graphs show triplicates of independent qRT-PCRs for each gene. Upper graphs: amplifications in which all genes were reverse-transcribed and amplified together in the first RT-PCR. Lower graphs: amplifications in which each gene was reverse-transcribed and amplified separately in the first RT-PCR. The histograms compare PCR efficiency in the two conditions. (**C**) Examples of differences between population-based readouts and single-cell readouts. A mature monoclonal CD8 T cell population was sorted and tested on the same day for expression of two different genes (genes a and b). Upper graphs: cells were sorted at 100 cells/well, in order to mimic population studies in which only average gene expression can be evaluated. The results demonstrate that amplifications of genes a and b have the same efficiency and that both genes are expressed to the same extent. On the basis of this data, one would conclude that the two genes are similarly expressed in this cell population. Lower graphs: each well received a single cell that was tested for the expression of genes a and b. In contrast to the population studies, these single-cell studies reveal that the respective expression levels of genes a and b genes are very different: gene b is expressed at low levels by all cells and gene a is expressed at high levels by only 10% of the cells.

The approach used to determine these parameters is summarized in **Fig.** **1A** and described in detail in the Methods section. To be used for all possible gene combinations, the approach requires strict rules for primer selection and amplification steps. We have thoroughly described and validated these rules previously [22]. For each particular set of genes studied, the primers must have two additional properties. Firstly, to enable quantitative comparisons of the expression of different genes, all individual amplifications must have the same efficiency. Secondly, given that the initial PCR amplifications are performed in the presence of 18 individual primers, it is essential to show that neither the primers nor the generated amplicons compete with one another. The primer combinations used in the present experiments fulfilled both criteria; all the amplifications of individual genes had the same efficiency (as shown by the parallel slopes for each individual amplification; **Fig.** **1B**) and did not compete with one another (as shown by similar amplification efficiencies when amplifications were performed either for a single gene or for all genes simultaneously. **(** **Fig.** **1B, C** **)**. Simultaneous compliance with these two criteria enabled us to quantify the absolute number of mRNAs coding for each gene in each individual cell, relative to a simultaneously amplified standard with a known mRNA copy number. Moreover, negative results can be accepted with confidence because by using two independent criteria, we have already demonstrated that the method can reliably detect as few as two mRNA copies per cell [22].

When interpreting the results, it is essential to understand the major differences between the novel information provided by the present approach and that obtained using gene expression arrays. Arrays are easy to perform and enable almost the entire mouse genome to be screened. In contrast, the single-cell method is laborious and only allows around 20 known genes to be screened each time. However, our present and previous results demonstrate that single-cell assays provide fundamental information that cannot be obtained in array studies, and vice-versa. By covering the entire genome, arrays have a fundamental role in identifying genes that were previously not known to be involved in a particular differentiation process. Once these genes have been identified, arrays do not provide much information on gene expression behavior and are frequently misleading, since they can only identify the average level of gene expression in a cell population. An example of this problem is shown in **Fig.** **1C**, where the same two genes (**a**, **b**) are studied simultaneously in the same cells at the population level (100 cells together) or at the single-cell level. When studied at the population level, genes **a** and **b** appear to be expressed similarly, since their amplification curves overlap. In contrast, when studied at the single-cell level, the expression is found to be totally different: gene **b** is expressed moderately by all cells, whereas gene **a** is strongly expressed but only by 10% of the cells. This example highlights another major limitation of arrays; they cannot identify the presence of subpopulations of cells with different characteristics. Since only average gene expression is evaluated, it is not possible to determine whether an increased signal is due to (i) a higher frequency of expressing cells, (ii) the same proportion of cells expressing higher levels of the gene or (iii) a loss of expression in most cells and compensatory expression of very high levels by a small subpopulation. The same reasoning applies to a reduction in signal or to an apparent absence of change in gene expression: i.e., all possible combinations of frequency vs. expression level variations or general expression vs. restricted expression with potentially major biological significance cannot be characterized in array studies. Importantly, arrays cannot determine gene associations at the single-cell level; since most biological processes (particularly during differentiation) require the co-expression of several genes, single-cell analysis is the only method capable of identifying interactions between different molecules.

Lastly, to simplify the monitoring of complex data, we divided gene expression profiles/interactions into those leading to T cell commitment and those involved in αβ/γδ lineage specification. The overall gene expression profiles of all thymocyte subpopulations are shown in **Fig** **S2.**

### Towards T cell commitment: Interactions between T master regulators

**Figure 2:**
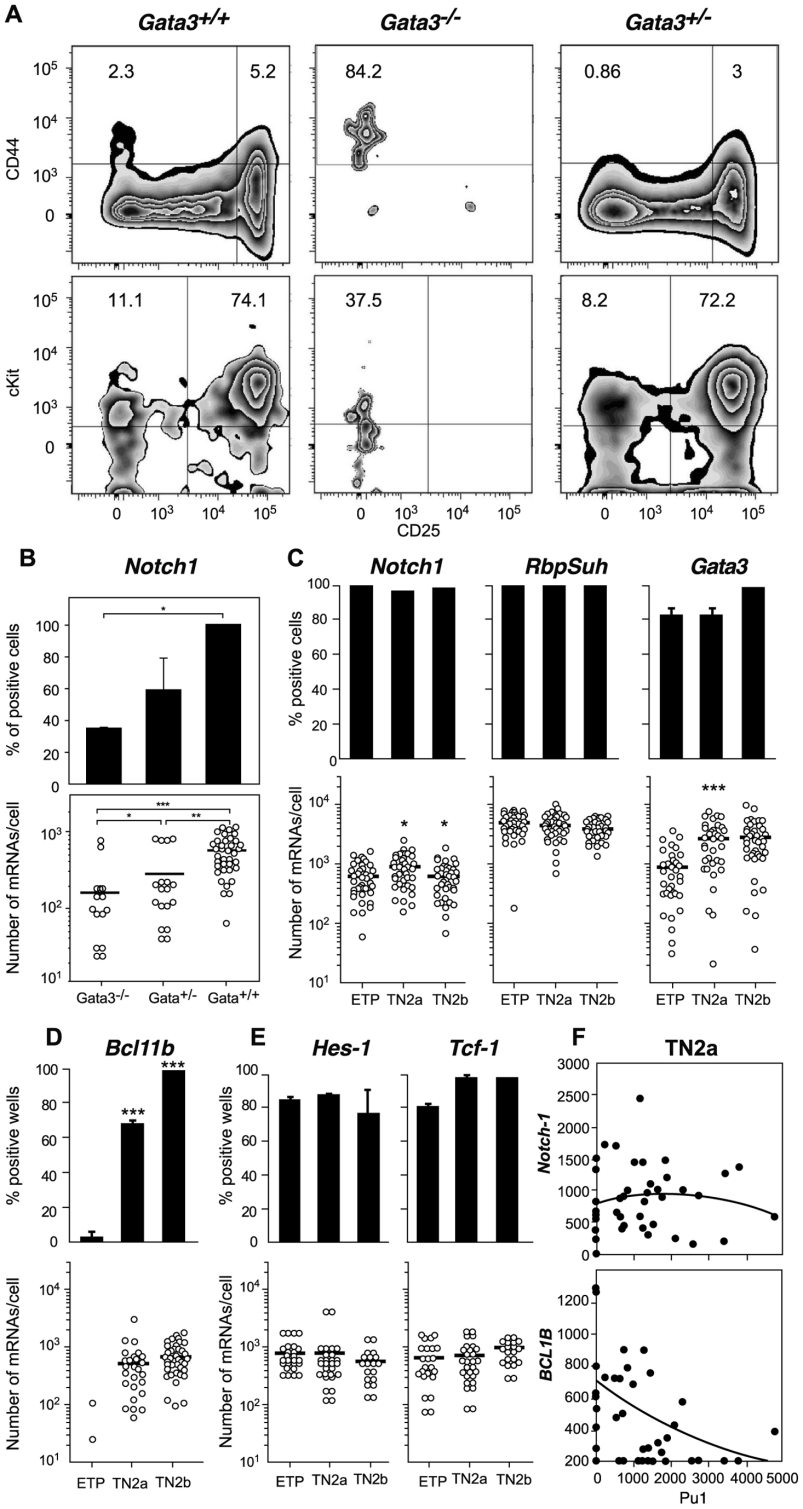
Transcription factor interactions during T cell commitment . **(A, B).** CD45.2^+^ 15-day fetal liver cells from *Gata3^+^/+* (left) *Gata3^−^/−* (middle) and *Gata3^+^/−* (right) mice were injected into sublethally irradiated CD45.1 mice. The CD45.2^+^ cells present in the thymus one month later were studied. (**A**) the phenotype of TN thymocytes. Upper graphs show CD44/CD25 profiles and lower graphs show c-Kit expression in gated CD44^+^ cells. (**B**) *Notch1* expression in TN1 cells from these chimeras. (**C**–**E**) Expression of different TFs during T cell commitment. B–E: Upper graphs depict expression frequencies (the proportion of positive cells) and lower grafts show the mRNA expression level in each individual cell (represented by a dot) from three independent experiments. Expression-negative cells and cells expressing fewer than 10 mRNAs/cell are not shown. Bars represent mean expression levels. Statistical analysis was performed using Fisher's exact test for expression frequencies and a Mann-Whitney rank sum test for expression levels. Asterisks correspond to a comparison of the population of interest with the population in the previous transition stage: * p<0.05, ** p<0.01 and *** p<0.001. **F.** The co-expression of the different genes was studied in forty individual cells. Each dot represents an individual cell, plotted simultaneously for the number of *Pu1* mRNA molecules on the X axis and the number of mRNA molecules coded as either Notch1 (upper graph) or Bc11B on the Y axis (lower graph). The correlation between the respective expressions of each pair of genes was studied in a Goodman-Kruskal gamma test, which assesses the correlation's significance (via a p-value) and nature (via the gamma coefficient, which is negative for a negative correlation and positive for a positive correlation). A polynomial trend curve is shown for each correlation.

Deletion of *Notch1* or inhibition of Notch1 signaling in hematopoietic stem cells blocks T cell development [2]. Similarly, *Gata3* deletion results in the absence of mature T cells and the generation of only a small number of ETPs [3]. With a view to determining why a Notch1 or Gata3 deficiency might induce similar blocks in TN cell differentiation, we studied the generation and properties of TN cells in chimeras injected with fetal liver cells from Gata3-competent, Gata3-deficient or haplo-insufficient donors (**Fig.** **2A**). Competent cells reconstituted TN populations as found in normal thymi, whereas Gata3*^−/−^* precursors only generated rare CD44^+^CD25^−^ cells expressing low c-Kit levels [3]. Furthermore, we found that Gata3*^+/−^* precursors had normal c-Kit expression levels but were yet poorly able to generate TN1 and TN2 populations. The low number of TN1 cells recovered from these chimeras prevented us from performing extensive single-cell studies. However, in the 27 individual Gata3^−/−^ TN1 thymocytes recovered in two independent experiments, Notch1 expression frequencies were much lower (**Fig.** **2B**). Moreover, the average Notch1 transcription rate in the rare Notch1*^+^* cells was 115 mRNAs/cell (compared with 750 mRNAs/cell in controls). It could be argued that the *Gata3*-deficient TN1 thymocytes were not directly comparable to ETPs, since their c-Kit expression level is much lower. Although Gata3^+/−^ TN1 cells expressed normal c-Kit levels (**Fig** **2A**), the proportion of cells expressing Notch1 was much lower than in Gata3^+/+^ cells: the Notch1 expression levels in positive cells averaged 256 copies/cell (vs. 750 copies/cell in controls) (**Fig** **2B**). In contrast, the expression level of the Notch1 cofactor RbpSuh did not change during these stages (**Fig.** **2C**). These findings demonstrate a dose-dependent role of Gata3 in sustaining Notch1 expression. It is likely that these mutual interactions are involved in the TN1 to TN2b transition and they may explain why the ablation of the Notch1 or Gata3 gene induces a TN1 differentiation block. Gata3/Notch1 interactions may also be required for the induction of Bcl11b. This TF is detected in TN2a cells when the concentrations of both Gata3 and Notch1 peak (**Fig.** **2D** **).** In contrast, the expression levels of Hes-1 and Tcf-1 do not change significantly in early TN populations **(** **Fig.** **2E** **).** Comparison of non-T-committed TN2a cells and fully T-committed TN2b populations showed major differences in the co-expression of these TFs. Only 50% of TN2a cells co-expressed Notch1+ Gata3+ Bcl11b + TcF-1, whereas all individual T-committed TN2b cells (**Fig.** **S1** co-expressed these genes **(** **Fig** **2** **C–D).**

### Interactions between T-lineage and non-T-lineage master genes

T cell commitment is associated with a decline in the expression of non-T master genes. The proportion of Gfi1b^+^ cells in the TN2b populations decreased but Gfi1b expression levels were maintained **(Fig.** **S2C).** The decline in Pu1 involved a drop in transcription frequencies, transcription rates **(Fig.** **S2C)** and protein expression (not shown). With a view to observing possible influences of T master genes on the expression of non-T master genes, we studied the co-expression of these two classes. We did not find any correlations between Notch1 **(** **Fig.** **2F** **)** or Gata3 (not shown) co-expression or expression levels on one hand and the co-expression of Pu1 on the other. In contrast, there was a very significant inverse correlation (p<0.0005, γ coefficient  = −0.34) between the expression/expression levels of Bcl11b and those of Pu1 **(** **Fig.** **2F** **).** Our results indicate that Bcl11b is the only of the nine TFs studied that can potentially be involved in the down-regulation of Pu1 expression.

It has been reported that Gfi1b down-regulates Gata3 expression in tumor cells [24]. We did not find any evidence of this role in normal TN thymocytes, since the TN2a population which expressed the highest levels of Gata3 (**Fig. 2C**) also had the highest proportion of Gfi1b-expressing cells (**Fig.** **S2C**).

In summary, the gene co-expression patterns and interactions described here indicate a fundamental, dose-dependent role of Gata3 in the maintenance of Notch1 expression. The interactions between Notch1 (which induces Gata3 expression [25]) and Gata3 (which has a dose-dependent effect on Notch1 expression levels) suggest that a positive feedback loop promotes and sustains T cell commitment. When individual thymocytes had become fully T-cell-committed (at the TN2b stage), they all co-expressed Notch1 + Gata3+ Bcl11b + Tcf1. This commitment did not require the extinction of non-T master genes, which only occurred in TN3 thymocytes (**Fig.** **S2C**).

### Towards αβ/γδ lineage commitment: Single-cell differentiation of TN populations in OP9DL4 cultures increases plating efficiency and reveals a new time course for lineage specification

To better characterize αβ/γδ lineage commitment, we sought to establish single-cell approaches to correlate a cell's differentiation potential with its ability to express the various components of the pre-TCR.

To this end, we optimized Notch1 signaling by performing single-cell cultures in the presence of the OP9 cell line transfected with DL4 (Notch1's native ligand in the thymus) [23]. Since we were plating individual cells from different TN subsets with different time courses for differentiation, each culture was checked every day for growth and small samples were taken for phenotyping. This strategy ensured that the cultures that we classified as being committed to one of the lineages never generated T cells of the other lineage at different time points during culture.

**Figure 3:**
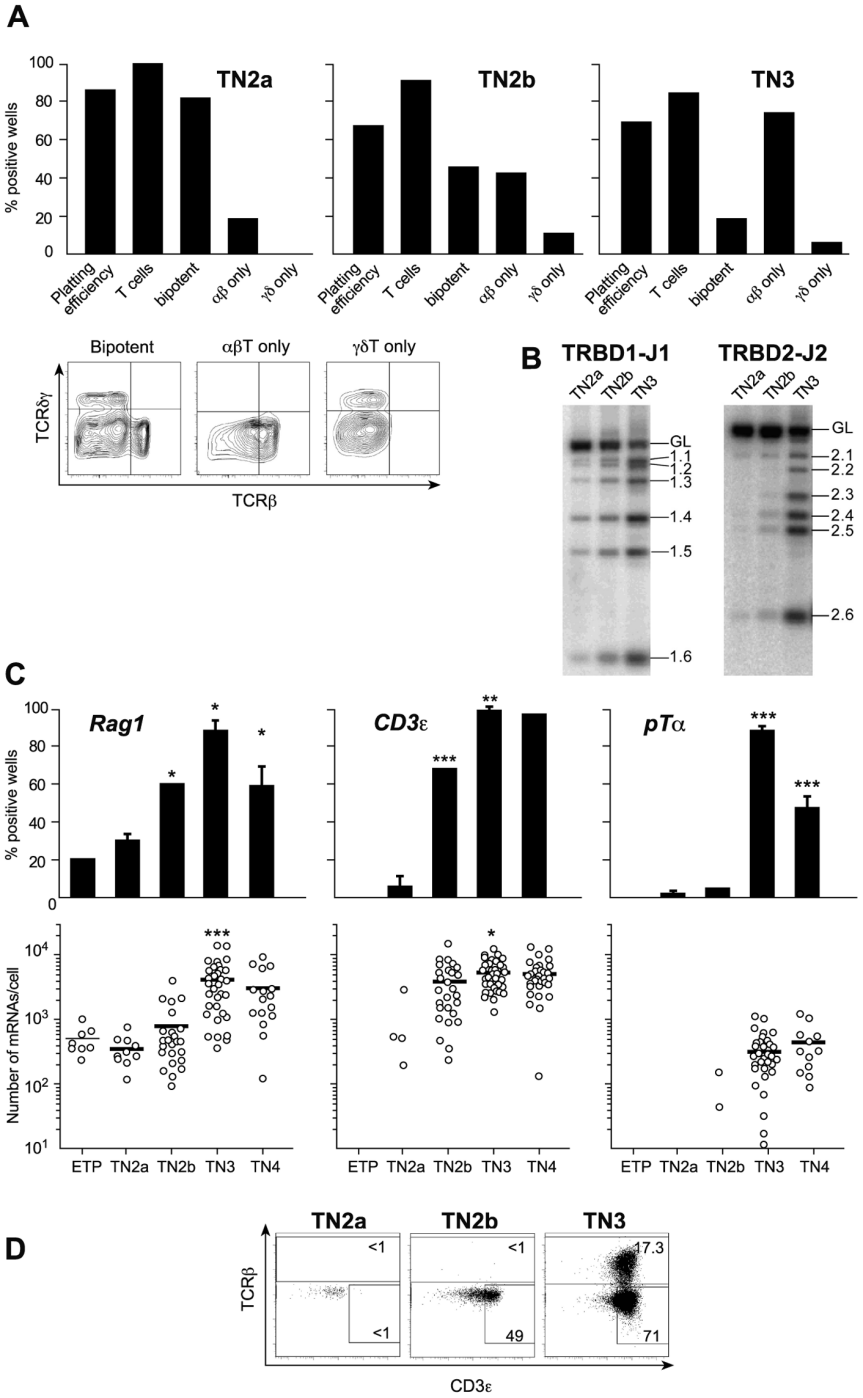
Differentiation potential of TN populations. A . Individual thymocytes were cultured with the OP9-DL4 cell line and sampled at different time points after plating. The depicted results are from 14-day cultures, since the T cell phenotype did not change after that time point. Plating efficiency: the percentage of plated wells in which progeny were detected; % T cells: the percentage of positive wells containing T cells. Bipotent, TCRαβ^+^-restricted and TCRγδ^+^-restricted cells were identified according to the dot plots shown below. **Please note**: in positive wells in which T cells were not detected, the average clone size was 100 cells/well; in both bipotent and TCR-αβ−only cultures, clone sizes averaged 2×10^5^ cells/well; in γδ−only cultures, average clone sizes were 5×10^4^ cells/well. Results are pooled from three independent experiments, each of which gave equivalent results. **B** Expression of different components of the pre-TCR in TN populations. *TRB* locus DJ rearrangements. **C**. *Rag1*, *CD3ε* and *pT*α expression in individual cells. Upper graphs show expression frequencies determined in 80 individual cells. Lower graphs show the number of mRNA molecules expressed by each individual cell studied (n = 40 cells). Each positive cell is represented by a dot, negative cells are not shown and bars represent mean expression values. Statistical analyses were performed as described in Fig 2. **D**. Intracellular TCRβ and CD3ε expression in different TN subsets in one representative experiment of four independent experiments with equivalent results.

When compared with single-cell cultures with OP9DL1 cells [12], single-cell differentiation in OP9-DL4 cultures yielded a major increase in plating efficiency (from 52% up to 84% for TN2 cells and from 24% up to 70% for TN3 cells (**Fig.** **3A**) and provided new knowledge on the time course of lineage specification. Although the majority of the TN2a progeny were bipotent, 18% (on average) were αβ committed. In TN2b, bipotent cell frequencies declined to 48% because the remaining cells were already committed to the αβ (≈42%) or γδ lineage (≈10%) (**Fig.** **3A**). The transition to TN3 did not increase the proportion of cells committed to the γδ lineage, whereas the percentage of TN3 cells generating exclusively TCR-αβ cells rose to 72%. In contrast to previous claims that all TN3 cells are lineage-committed [12], we found that an average of 20% of the individual TN3 thymocytes were still capable of generating both TCR-αβ and TCR-γδ cells. The presence of this significant, bipotent TN3 population has important implications for the interpretation of studies of signal strength in αβ/γδ lineage specification, as discussed below. We found that bipotent and αβ-committed cells generated higher cell yields (about 2×10^5^ cells/culture at day 14) than γδ-committed single cell cultures did (about 2×10^3^ cells/culture at day 14).

It is known that commitment events are frequently accompanied by proliferative bursts, as described in TN4 populations after the passage through the pre-TCR/γδ check point [26]. We also found that the fully T-cell-committed TN2b population had a high proportion of BrdU^+^ cells (**Fig.** **S3**) – showing that TN populations undergo an additional, major proliferative burst associated with T lineage commitment.

### Quantification of thymocyte populations expressing the different components of the pre-TCR

To study the role of the pre-TCR in αβ lineage commitment, we investigated the putative correlation between the differentiation potential of TN single cells and the latter's expression of the various elements of the pre-TCR (**Fig.** **3B**–**D**). Although 18% of TN2a cells were αβ-committed, none were found to express the pre-TCR. Only 30% of the cells expressed Rag1, and Rag1 expression levels in these cells were low. TCRBDJ rearrangements were quite rare and the TCR-β chain, CD3ε and pTα were not expressed (**Fig.** **3B**–**D**). TN2b populations were more mature, since the frequency of Rag1 expression was substantially higher and CD3ε was suddenly switched on. However, these genes were expressed at relatively low levels – explaining the very low frequency of TCRB locus rearrangements. The TCRBDJ locus was mostly in a germ-line configuration and the TCR-β chain was not expressed (**Fig.** **3B**–**D** **)**. Moreover, pTα was virtually absent, in agreement with a publication [27] but in contrast to the latest report by the same group [28]. We conclude that although 43% of TN2b cells were committed to the αβ lineage, none carried the pre-TCR.

Surprisingly, a large proportion of TN3 cells could not express the pre-TCR either. Although virtually all TN3 thymocytes expressed Rag1, the latter's mRNA copy number/cell increased tenfold, CD3ε became ubiquitously expressed and PTα was suddenly induced in the majority of cells (**Fig.** **3C**), most TN3 cells had yet to complete their TCRB rearrangements. DJ germ-line bands were clearly detected **(** **Fig.** **3B** **)**, which contrasts with the situation in mature TCR-αβ cells [29]. Although 73% of TN3 cells were committed to the αβ lineage, only 17.3±3% expressed the TCR-β chain and therefore had the potential to express the pre-TCR (**Fig.** **3D**). With a view to determining the proportion of αβ-committed cells expressing the pre-TCR, we correlated our frequency data (**Fig.** **3**) with the mean proportion of each TN subpopulation **(** **Table** **1** **).** This calculation showed that on average, 83% of αβ-committed cells did not express the pre-TCR. Our data clearly show that αβ-committed TN thymocytes not expressing the pre-TCR cannot be regarded as an outlier population that does not follow the general rules governing αβ lineage choice. Indeed, the vast majority of αβ-committed cells failed to express the pre-TCR.

**Table 1:** Evaluation of committed progenitors: all values were obtained from figures within thymic (TN) or committed (Com) progenitors.

	% TN	%αβ-Com (in each population)	% αβ-Com (in TN)	% Pre-TCR^+^ (in each population)	% Pre-TCR^+^ (in TN)
TN2a	0.4	18	0.072	0	0
TN2b	1.6	42	0.672	0	0
TN3	46	72	33.12	17.3	5.73
		Total	33.864	Total	5.73
αβ-Committed	TN:	%Pre-TCR^−^	83		
		%Pre-TCR^+^	17		

### From TN3 to DP: changes induced by the transition through the pre-TCR/γδ check-point and TN4 heterogeneity

The precise point at which full αβ/γδ lineage commitment takes place is still subject to debate. Some researchers have suggested that when TN3 cells express CD27, they are all lineage committed and ready to pass the pre-TCR/γδ check-point [30]. However, these studies were performed at the population level and thus could not rule out the presence of some non-committed cells within the cell set. Indeed, some researchers have suggested that full commitment occurs much later [18].

**Figure 4:**
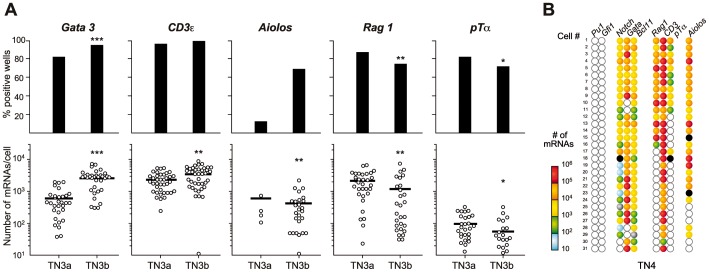
Gene expression in TN3a and TN3b populations. A . TN3a and TN3b populations were sorted as single cells and tested for the expression of each gene. Upper graphs show expression frequencies (determined in 80 individual cells). Lower graphs show the number of mRNA molecules expressed by each individual cell studied (n = 40 cells), depicted as described in Fig. 1. **B**. Gene co-expression patterns in individual TN4 cells. Each horizontal row corresponds to the same (numbered) cell. Each column shows the expression of a different gene, with the number of mRNA molecules/cell represented according to the adjacent color log scale. Empty symbols represent cells not expressing that particular mRNA (i.e. fewer than two mRNA molecules per cell). The black symbol corresponds to a positive cell in which quantification was not performed.

In the TN3a-to-TN3b transition, Notch1 expression did not change (data not shown), whereas Gata3, Cd3ε and Aiolos expression frequencies and/or expression levels increased and those of Rag1 and pTα declined (**Fig.** **4A** **)**. Surprisingly, two very different cell subsets stood out within the TN4 population (**Fig.** **4B** **)**. Approximately 60% of the TN4 cells had much the same characteristics as TN3a thymocytes (i.e. relatively high levels of Notch1, Rag1 and pTαexpression). In the remaining cells, Notch1 and pTα were either downregulated or not expressed (as we had also found in DP cells – data not shown), Rag1 was absent and Gata3 expression levels were upregulated. This more mature population might correspond to the TCR-γδ precursors that reportedly emerge at this differentiation stage [20]. However, 80% of these cells expressed Aiolos (**Fig.** **4B**), which is supposedly a marker for αβ lineage-committed cells [27]. Although we lack an explanation for the clear dichotomy in TN4 populations, our results reveal a significant degree of heterogeneity in late TN differentiation.

## Discussion

Several approaches have been used to study early thymocyte differentiation. Here, we developed a new *ex vivo* approach that enabled us to describe how TN thymocytes co-express a defined set of genes under physiological conditions.

Both Notch1 and Gata3 are known to have a major role in the survival and differentiation of ETPs [25]. It was shown that Notch1 induces Gata3 expression, since introduction of Gata3 before the activation of Notch1 signaling fails to promote T cell development [25]. However, Gata3′s role in early TN differentiation remained unclear; although the ablation of Gata3 induces a major block in TN1 differentiation, the TF was found not to be essential for the generation of thymocyte progenitors in the BM. Furthermore, Gata3 does not appear to influence thymus colonization and neither blocks ETP division nor promotes ETP apoptosis [3]. We found that Notch1 expression is virtually abrogated in c-Kit^low^, Gata3-deficient TN1 cells and is very low in c-Kit^+^ Gata3 haplo-insufficient ETPs. These findings indicate that the survival of ETPs and the latter's progression through the differentiation process depends on interactions between Notch1 and Gata3. Notch1 induces and regulates the expression of Gata3, which in turn has a fundamental, dose-dependent role in the maintenance of Notch1 expression levels. These interactions might create a positive feedback loop and might be required for induction of the Notch1 target gene Bcl11b. Indeed, we found that the respective mean mRNA copy number for Notch1 and Gata3 increased (by a factor of 3 and 5, respectively) in the ETP-TN2a transition (when Bcl11b is first expressed). In contrast, neither Notch1 nor Gata3 co-expression patterns are compatible with a direct, suppressive effect on the expression of non-T-lineage genes. Of the TFs studied here, only the Bcl11b expression patterns were compatible with this role (with a very marked inverse correlation with the co-expression of Pu1). Although this inverse correlation does not prove that Bcl11b down-regulates the expression of Pu1, there is other independent, supporting evidence. It has been shown that (i) Bcl11b ablation prompts the differentiation of thymocyte precursors into natural killer (NK) cells [4], [5], [6] and (ii) Pu1 has a major role in NK generation and differentiation [31].

We confirmed that full T cell commitment occurs in the TN2b population, as reported previously [28]. It is usually assumed that repression of non-T-lineage master genes must be complete before T cell commitment takes place. However, our results indicate that a mere reduction in expression of these genes is enough to allow full T cell commitment, since both Pu1 and Gfi1b are still being expressed in the T-cell-committed TN2b set. In contrast, a major difference between TN2a and TN2b relates to the co-expression of Notch1 + Gata3+ Bcl11b+ Tcf-1 in individual cells. Indeed, these four genes are co-expressed by only half the TN2a cells but by all individual T-cell-committed TN2b cells. It is possible that the co-expression of these 4 genes is required for full T cell commitment. Alternatively, the very high Gata3 expression levels found in TN2b cells may have a major role in T cell commitment, as we found in the extrathymic T-cell-committed precursors in BM-derived spleen colonies or in the nude spleen [32].

Once the TN2b populations had transitioned to the TN3 stage, non-T-lineage genes were totally silenced and T-cell-specific genes were turned on sequentially: Rag1 and CD3ε were turned on at the TN2b stage and then upregulated in TN3, pTα was turned on in TN3 only and the transcription rates of both Gata3 and Bcl11b declined. The study of TN single-cell differentiation potential in co-cultures with the OP9DL4 cell line enabled us to increase the plating efficiency considerably. Under these conditions, a comparison between each cell's differentiation potential and its expression of T-lineage-specific genes provided insights into the mechanism of αβ/γδ commitment. Firstly, by looking at the time course of events on the single-cell level, we found that lineage commitment may occur at very different phases in the TN ontogeny – ranging from TN2a to TN3 or possibly even later. Concerning mechanistic aspects, about 83% of TN cells became committed to the αβ lineage before they were able to express the pre-TCR. These results do not undermine the fundamental role of the pre-TCR, which is probably expressed in these cultures before TCR-αβ mature cells are generated. In fact, our results redefine the pre-TCR as a differentiation factor involved in the expansion of pre-committed thymocytes and the latter's differentiation into DP cells and TCR-αβ cells. This type of role fits best with the stochastic model, which separates commitment from differentiation and features an initial commitment phase that is induced by a particular combination of master genes. Further differentiation of committed cells would then occur once αβ/γδ-specific differentiation factors are expressed.

Although not all the plated single cells differentiated *in vitro*, the high observed T cell counts and αβ plating efficiencies contradict this hypothesis. It cannot be held that the αβ lineage-committed TN2b cells detected in clonogenic assays (84% of the TN2b set) were the progeny of precursors already expressing the pre-TCR, since none of the TN2b cells expressed TCR-βand only 5% expressed low levels of pTα. Similar calculations for TN3 populations reveal that the proportion of lineage-committed cells (77%) was much higher than predicted on the basis of the proportion of precursors expressing the TCR-β chain (17%, on average).

These results do not rule out a fundamental role of the pre-TCR in the generation of TCR-αβ cells but do redefine the pre-TCR's role as a fundamental factor in the expansion and differentiation of pre-committed cells into TCR-αβ cells. It is likely that commitment to differentiation into the γδ lineage also precedes the expression of TCR-γ. In fact, TCR-γ rearrangements in TN2 populations are very rare (L. Peaudecerf, P. Pereira and B. Rocha, unpublished data) and it has been reported that TCR-γ protein cannot be detected in TN2 cells [33].

Our data showed that commitment to αβ/γδ lineages precedes the expression of the TCRγδ or the pre-TCR in the vast majority of thymocytes; this might appear to be incompatible with previous experiments in which signal strength was found to have a role in lineage specification. In these experiments, thymocytes from γδ TCR transgenic mice were shifted to DP differentiation by a reduction in the TCR-γδ signal strength [16]. However, the thymocyte population comprised both committed and non-committed cells. Under these circumstances, it is not possible to say whether changes in signal strength induced lineage commitment or, in contrast, were merely required for the differentiation of precursors into TCR-γδ^+^ cells. The results of other single-cell experiments suggested that signal strength could modify cell fate relatively late in the thymocyte ontogeny and that lineage choice was flexible until TN thymocytes had differentiated into either DP cells (for the αβ lineage) or CD24^−^ TCR^+^ CD4^−^CD8^−^ populations (for the γδ lineage). However, only 4% of the plated single cells grew in culture and deviated to alternative lineages [18]. Therefore, instructive signals were only directly effective in a very small number of TN cells. Lastly, it was also shown that the presence of the pre-TCR reduced the proportion of TCR-γδ cells expressing out-of-frame TCRβ rearrangements; this observation suggested that pre-TCR signaling deviated TN cells to the TCR-αβ lineage [19]. Again, both the low number of characterized cells (21 in pre-Tα-deficient mice) and the choice of selection criteria could have biased these conclusions. This evaluation assumed that all TCR-γδ cells should have rearranged both TCRB alleles and thus excluded about 60% of the cells studied (i.e. those having undergone no TCRB locus rearrangements or only mono-allelic rearrangements). However, taking account of these excluded cells would considerably change the prediction of out-of-frame rearrangements. Lastly, one can argue that the differentiation potential of thymocyte precursors in OP9-DL4 cells does not correspond to lineage commitment because the latter requires the presence of other signals. In this hypothesis, commitment can only be demonstrated when none of the signals introduced into these cultures modified the fate of the plated cells. This approach confuses commitment signals with differentiation signals. In fact, later-stage signals (such as TCR-γδ pre-TCR signal strength) may only be required for differentiation. For cells committed to a particular lineage, only those expressing the differentiation factor corresponding to that lineage will differentiate. Although the introduction of differentiation factors may modify the proportion of cells committed to either lineage, this change may be only reflected by a reduction in the plating efficiency. Moreover, since all TN populations studied to date have been mixtures of lineage-committed and non-committed cells, we consider that commitment studies must be always performed at the single-cell level. Overall, the approach described here can be used to quantify the expression of the pre-TCR in non-manipulated single cells and assess the latter's differentiation potential. Given the limitations of currently available differentiation cultures (which do not enable commitment factors to be distinguished from differentiation factors), our study provides novel information on the respective impacts of stochastic and instructive signals on αβ/γδ lineage commitment. The literature data has been interpreted as suggesting that stochastic commitment only operates in a small proportion of thymocytes (a subpopulation of the rare TN2 set) and that the remaining cells follow instructive rules. Our present data suggest that stochastic commitment operates in the vast majority of TN cells and that only a small minority of TN thymocytes follows instructive rules.

We identified the TN differentiation stages at which αβ/γδ lineage commitment began and at which the vast majority of TN thymocytes became lineage-committed. However, we did not identify the differentiation step at which all individual thymocytes became αβ/γδ lineage-committed. Our data suggests that this process takes longer than proposed previously [12]. We found that 20% of the TN3 cells were still bipotent. Although it has been suggested that all TN3b thymocytes are lineage-committed [30], other data revealed the presence of TCR-γδ^−^ TCRβ^−^ cells within this population [18]. Moreover, we found that TN4 populations were heterogeneous; one fraction was still expressing the high levels of Rag1, Notch1 and pTα characteristic of TN3a cells, whereas another fraction did not express Rag1 and showed the downregulation of Notch1 and pTα characteristic of DP and mature thymocytes. The latter fraction might correspond to the TCR-γδ precursors that reportedly emerge at this differentiation stage [18]. However, this hypothesis was contradicted by the cells' co-expression of Aiolos, which is thought to demonstrate αβ commitment [30]. The considerable heterogeneity seen in late TN populations indicates that further studies will be required to characterize this subset.

## Materials and Methods

### Mice

Triple-negative populations were obtained from 6- to 8-week-old B6 mice purchased from Centre d'Elevage R. Janvier (Le Genest St Isles, France). Thymocytes from Gata-3 deficient mice were obtained by injecting Ly5.2 fetal liver precursors into sublethally irradiated Ly5.1 hosts [34].

### Ethics statement

All experiments were carried out in accordance with the guidelines of the French Ministry of Agriculture, under a personal license (number 75–1026). No approval was necessary (government decree number 2013–118)

### Antibodies

The monoclonal antibodies (mAbs) used for cell sorting were obtained from BD Pharmingen (San Diego, CA, USA). For cell sorting, Lin^+^ thymocytes were first depleted by magnetic sorting using TER119, Gr1 (RB6-8C5) and CD8 rat mAbs, sheep anti-rat IgG-conjugated beads and sheep anti-mouse IgG-conjugated beads (Dynabeads M-450; Dynal Biotech A.S., Oslo, Norway). Triple-negative cells were further labeled with antibodies against lineage antigens (Mac-1, NK1.1, TCRαβ, TCRγδ, CD8α and CD19), CD25, CD44, c-Kit and CD27. Single cells from each TN subpopulation were collected in individual PCR tubes (containing 5 µl of 0.1% PBS-DEPC using a FACSAria I equipped with an automatic cell deposition unit (BD Biosciences, San Diego, CA, USA)) and stored at −80°C.

Intracellular staining for TCRβ and CD3ε proteins was performed with the Cytofix/Cytoperm Fixation/Permeabilization kit (BD Biosciences), according to the manufacturer's instructions. To target cells in S-phase, mice were injected intraperitoneally with BrdU (BD Biosciences) (as described previously [26]) and studied 60 minutes later. Incorporation of BrdU was performed according to the manufacturer's instructions.

### Quantitative single-cell RT PCR

These experiments were performed as described in the previously published validation study [22]. Briefly, single cells from each TN subpopulation (Fig. S1) and the TN3a TN3b populations (data not shown) were collected in individual PCR tubes (containing 5 µl of 0.1% PBS-DEPC using a BD FACSAria I equipped with an automatic cell deposition unit (BD Biosciences)) and stored at −80°C until use. The cDNA was prepared by gene-specific RT with the specific 3′ primers for all the genes studied and for a housekeeping gene. This step was followed by a 14-cycle amplification in the presence of specific 5′ primers for all genes. The PCR products from each single-cell amplification were then aliquoted into wells in which the expression of each gene was quantified separately by using semi-nested qPCRs. Slope values for the exponential PCR phase were determined using the Sequence Detector System Software (version 2.2, Applied Biosystems Inc., Branchburg, NJ, USA). The process for primer selection is indicated as indicated. Gene sequence data were taken from the Ensembl (http://www.ensembl.org) and NCBI nucleotide databases (http://www.ncbi.nlm.nih.gov/entrez/). Primers were designed manually, according to the strict rules that we have described previously. Briefly, 3′ and 5′ primers were chosen from different exons (in order to avoid genomic amplification). To obtain similar amplification efficiencies for all mRNAs, we designed 20-base-pair (bp) primers for similar-sized fragments and with similar melting temperatures (T_m_) and guanine-cytosine contents (around 50%). To avoid primer competition, primers and potential amplicons must not cross-hybridize. Primer compatibility was first assessed using Amplify 1.2 software (http://engels.genetics.wis.edu/amplify) and then tested experimentally in the competition experiments described below. We frequently found competition that had not been predicted by the Amplify 1.2 software, and so other primer pairs had to be selected and tested. The primers used in this study are listed in **Table** **S1.** The generation of a standard cDNA with a known number of mRNA molecules is as indicated. To allow absolute quantification of the number of mRNA molecules present in each cell, we prepared a standard cDNA with a known number of mRNA molecules and that could be amplified with the same efficiency as in the other RT-PCR reactions. To this end, we used *Cmyb* cDNA extracted and amplified from Lin-ckit + Sca1+ (LSK) BM cells. This cDNA was harvested from a 1.5% agarose ethidium bromide gel and then purified using the Wizard SV Gel/PCR Clean-up System (Promega Corporation, Madison, WI, USA). The purified cDNA was then assayed using the Picogreen incorporation method (Molecular Probes Inc., Eugene, OR, USA), using an ABI PRISM 7900 HT Sequence Detection System (Applied Biosystems Inc.). The concentration of the purified *Cmyb* cDNA was then determined with respect to a dsDNA from plasmid (Molecular Probes Inc.). The validation of the different steps in the RT-qPCR reaction is as indicated. This method requires strict rules for primer selection and the simultaneous validation of several different steps, as described in detail previously [22] and below. Firstly, we had to prove that the efficiency of reverse transcription (RT) was the same for each gene studied. Secondly, primers have to be selected so that (i) they have the same efficiency and (ii) neither the primers nor the amplicons they generate compete with one another (since multiple primers are present in the first PCR reaction). While selecting primers with similar efficiency is usually an easy task, simultaneously preventing competition usually requires the selection of primers from very different parts of the gene. These different locations prevented us from using PolyA reverse transcription, which favors 3′ RTs and thus could differ in its efficiency as a function of primer location. We preferred to use gene-specific RT and have already shown that the RTs of each gene had the same efficiency. Secondly, this single-cell RT-qPCR comprises two independent steps – first an RT-PCR and then a semi-nested qPCR. We thus had to determine the number of amplification cycles in first RT-PCR reaction that allowed the detection of a very low mRNA copy number without reaching saturation (even when transcription rates were very high). To evaluate this parameter, we amplified different, known numbers of synthetic RNA molecules by varying the number of amplification cycles in the first PCR. We found that the use of 14 cycles enabled us to detected as few as 10 RNA copies and did not cause saturation (even when 108 RNA molecules were amplified). However, since the amplifications of synthetic RNA and cellular RNA may differ, we further refined our evaluation of the method's sensitivity. Rather than amplifying mRNA, we looked at whether our amplification procedures could detect a given gene in each individual cell. In the absence of RT and after proteinase K treatment, our primers and amplification procedures indeed detected the gene in all single-cells [22]. These data proved that the method detected a copy number as low as two, since only two copies of a gene were present in the genome. It is noteworthy that all the primers used here are located in different exons. Hence, DNA amplification could not have biased our RT-PCR results because otherwise the PCR product would have contained the intron and would have been larger than the PCR product generated after mRNA amplification. In addition to these general rules (which apply to all gene combinations studied), it was also necessary to demonstrate that all amplifications of each simultaneously studied set of genes have the same efficiency and do not compete with one another. The primers used in this study complied with both these requirements: they all had the same efficiency (as shown by the parallel amplification curves) and did not compete with one another (since amplifications using a gene's primers alone gave the same results as when the primers for all the genes were present).

### OP9DL4 co-cultures

OP9 or OP9-DL4 cell lines were cultured in the presence of 1 ng ml^−1^ IL-7 and 5 ng ml^−1^ of the corresponding Flt3 ligand [12]. For the mass culture assays, 200 TN cells were cultured in 24-well plates. For clonogenic assays, individual cells were cultured in a 96-well round bottom plates. To prevent artifacts associated with different growth or survival rates, individual cultures were monitored for growth and small numbers of cells were sampled to identify the progeny at different time points after plating.

### PCR analysis of T cell receptor gene rearrangements

TCRBDJ and VDJ rearrangements were identified as described previously [35].

## Supporting Information

Figure S1 **The TN subsets studied.** (A) The TN compartment is shown on the basis of CD44 and CD25 distributions. Within the CD44^+^CD25^+^ window (R1), two populations are depicted as a function of the c-Kit intensity: c-Kit^hi^ (TN2a) and c-Kit^lo^ (TN2b) populations. Within the R2 window (CD44^−^CD25^+^), all cells are c-Kit^−^(TN3). (B) The differentiation potential of each subset, cultured in the presence of OP9 or OP9DL-4 stroma cells.
(TIF)

Figure S2 **Expression of master regulators during thymus differentiation.** Different Lin^−^TN populations were sorted as single cells and each was tested simultaneously for the expression of master regulator genes. Upper graphs depict expression frequencies, determined in 180 individual cells (ETPs) and 120 individual cells (TN2a and TN2b cells) collected in three independent experiments. Lower graphs show the number of mRNA molecules coding for each gene in each individual cell (n in total  = 30–40 cells). Each positive cell is represented by a dot, negative cells are not shown and bars represent mean expression levels. Differences in expression frequencies and gene expression levels were respectively determined using Fisher's exact test and a Mann-Whitney rank sum test. Asterisks correspond to the comparison of the population of interest with the preceding differentiation stage: * p<0.05, ** p<0.01 and *** p<0.001. Some of the results for ETP and TN2 populations are taken from Fig 2; the present Supplementary Figure further shows how gene expression changes over time in TN3 and TN4 sets.
(EPS)

Figure S3 **Division rates of thymocyte sets**. Mice were injected with BrdU and studied 60 minutes later (n = 3 mice/experiment; n = 9 mice in total). **Upper graphs** show the BrdU incorporation measured in one representative experiment. **Lower graphs** show the mean (±SD) values for the nine mice studied in three different experiments. The frequencies of BrdU^+^ cells in the animals were compared in a two-tailed T-test (* p<0.05, ** p<0.01 and *** p<0.001).
(EPS)

Table S1 (DOC)
