# Supplementary figures and images for: Single-Cell Analysis of Thymocyte Differentiation: Identification of Transcription Factor Interactions and a Major Stochastic Component in αβ-Lineage Commitment
Source: PLoS One. 2013 Oct 1;8(10):e73098. doi: 10.1371/journal.pone.0073098 (PMC3787938; doi:10.1371/journal.pone.0073098)

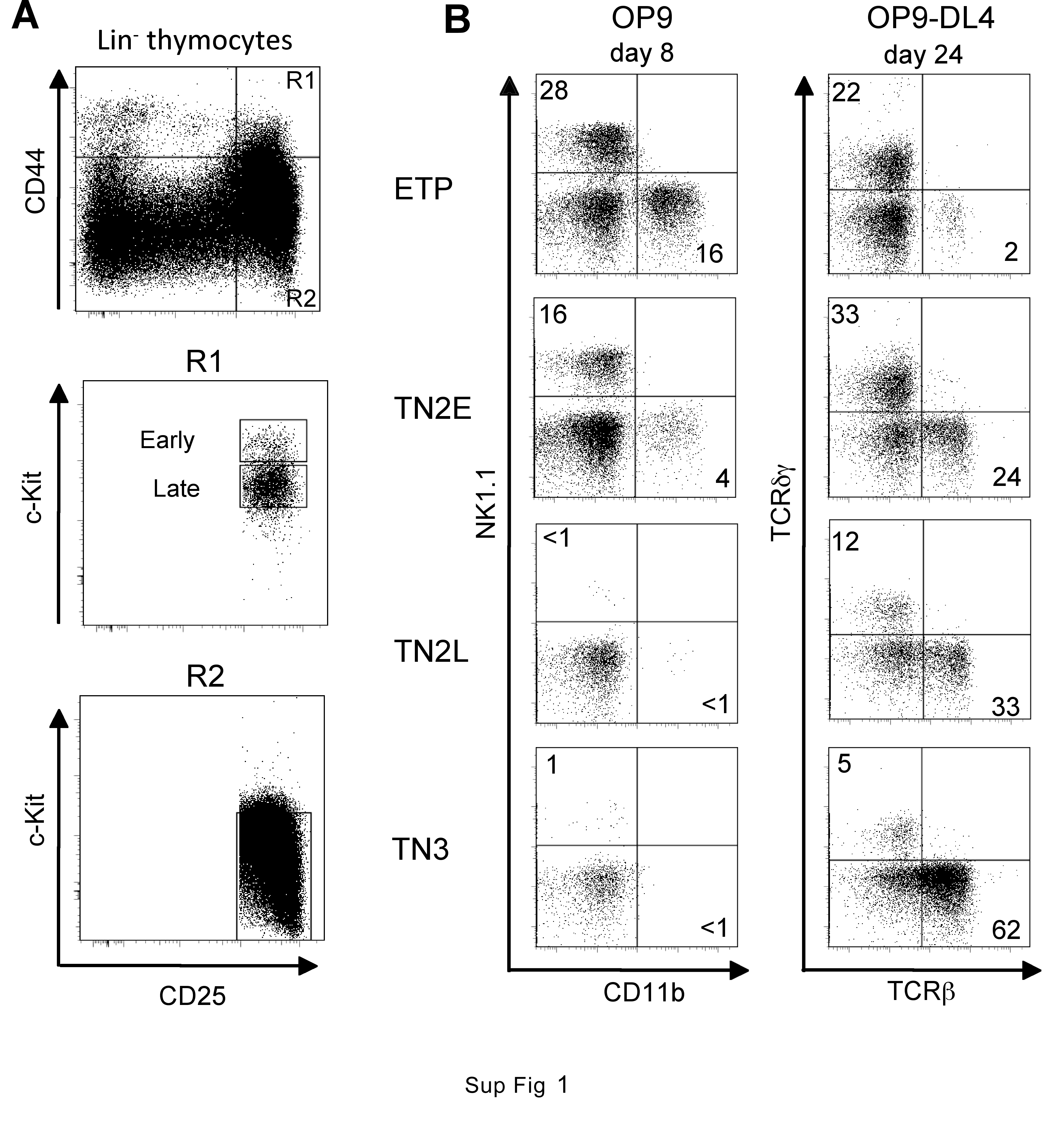

Supplement: Figure S1 — The TN subsets studied. (A) The TN compartment is shown on the basis of CD44 and CD25 distributions. Within the CD44+CD25+ window (R1), two populations are depicted as a function of the c-Kit intensity: c-Kithi (TN2a) and c-Kitlo (TN2b) populations. Within the R2 window (CD44−CD25+), all cells are c-Kit−(TN3). (B) The differentiation potential of each subset, cultured in the presence of OP9 or OP9DL-4 stroma cells. (TIF) [file pone.0073098.s001.tif]
