# Supplementary material for: Single-Cell Analysis of Thymocyte Differentiation: Identification of Transcription Factor Interactions and a Major Stochastic Component in αβ-Lineage Commitment
Source: PLoS One. 2013 Oct 1;8(10):e73098. doi: 10.1371/journal.pone.0073098 (PMC3787938; doi:10.1371/journal.pone.0073098)

**Table S1**: **List of primer sequences for used for single-cell genetic profiling**

Three primers were designed for each gene. A and C primers were sense, whereas B primers were anti-sense. B was used for RT, A and B for the first PCR and B and C for the second PCR. The sequences are 5’⇒ 3’.


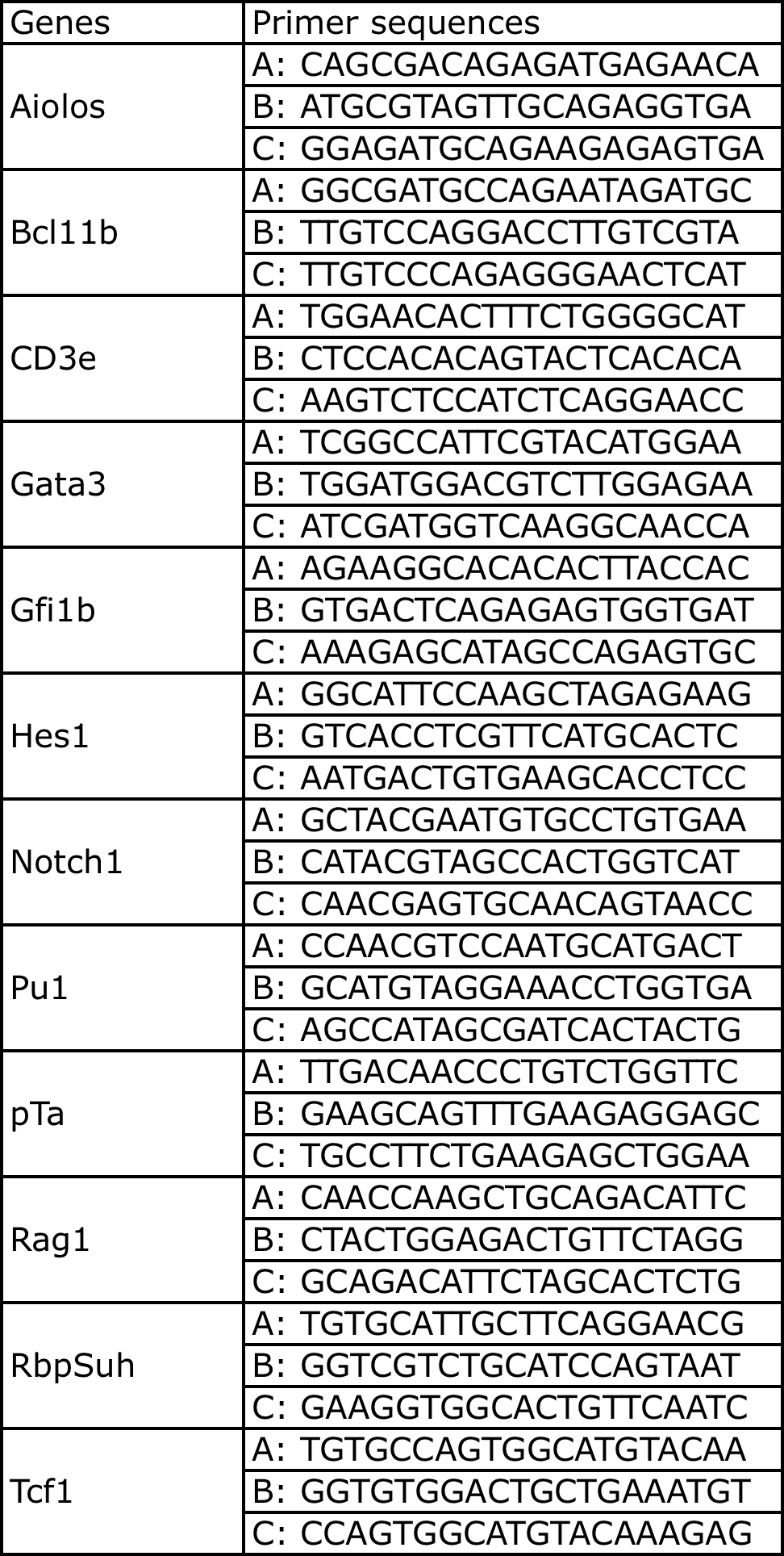

Supplement: Table S1 — (DOC) [file pone.0073098.s004.doc]
